# Plant and mammalian-derived extracellular vesicles: a new therapeutic approach for the future

**DOI:** 10.3389/fbioe.2023.1215650

**Published:** 2023-09-13

**Authors:** Ibrahima Mamadou Sall, Tabaran Alexandru Flaviu

**Affiliations:** Department of Anatomic Pathology, Faculty of Veterinary Medicine, University of Agricultural Sciences and Veterinary Medicine, Cluj-Napoca, Romania

**Keywords:** extracellular vesicle, therapeutics, microvesicles, nanovesicles, exosomes, apoptotic bodies, plant, mammal

## Abstract

**Background:** In recent years, extracellular vesicles have been recognized as important mediators of intercellular communication through the transfer of active biomolecules (proteins, lipids, and nucleic acids) across the plant and animal kingdoms and have considerable roles in several physiological and pathological mechanisms, showing great promise as new therapeutic strategies for a variety of pathologies.

**Methods:** In this study, we carefully reviewed the numerous articles published over the last few decades on the general knowledge of extracellular vesicles, their application in the therapy of various pathologies, and their prospects as an approach for the future.

**Results:** The recent discovery and characterization of extracellular vesicles (EVs) of diverse origins and biogenesis have altered the current paradigm of intercellular communication, opening up new diagnostic and therapeutic perspectives. Research into these EVs released by plant and mammalian cells has revealed their involvement in a number of physiological and pathological mechanisms, such as embryonic development, immune response, tissue regeneration, and cancer. They are also being studied as potential biomarkers for disease diagnosis and vectors for drug delivery.

**Conclusion:** Nanovesicles represent powerful tools for intercellular communication and the transfer of bioactive molecules. Their molecular composition and functions can vary according to their origin (plant and mammalian), so their formation, composition, and biological roles open the way to therapeutic applications in a variety of pathologies, which is arousing growing interest in the scientific community.

**Clinical Trial Registration:** ClinicalTrials.gov identifier: NCT03608631

## 1 Introduction

Extracellular vesicles (EVs), also called microparticles, microvesicles, or exosomes, are a heterogeneous group of phospholipids membrane-bound micro-to nano-sized biovesicles derived from eucaryotic and procaryotic cells. EVs are complex membranes composed of lipid bilayers containing mainly proteins and surface receptors (Table 1), protecting the EV content (soluble and genetic material, including miRNAs) from proteases and nucleases (Abels and Breakefield, 2016; Linares et al., 2017; Suharta et al., 2021). These membrane-demarcated particles are produced and released by the cells of all three realms of life and are diverse in their morphology, biogenesis, composition, and biological role (Gill et al., 2019).

**TABLE 1:** Representation of the liberation of the different types of mammalian-derived EVs.

Sub-types of Evs	Origin	Size (nm)	Commonly used markers	Ref
Exosomes	Fusion of the multi-vesicular body with a plastic membrane	30–150	CD63, CD9, CD81, Alix, Flotilline-1, ESCRT, and TSG 101	Alvarez-Erviti et al. (2011), Mathivanan et al. (2012), Lakhter and Sims (2015), Gurunathan et al. (2019)
Microvesicles	Plasma membrane burgeoning	50–1000	Caveolin, CD40 ligand, selin, flotillin-2, annexin V, and phosphatidyl-serine	Jayachandran et al. (2012)
Apoptotic bodies	Plasma membrane ballooning and cells in apoptosis	1000–5000	Annexin V, phosphatidyl-serine, and histones	Hristov et al. (2004)

The history of their discovery dates from 1868 when Charles Darwin first presented the concept of EVs. His theory was based on the idea that each cell type in the body generates small germs or gemmules (particles), permitting communication and transfer of hereditary information with other cell types. He also suggested that the composition of the granules could be modified by the environment, reflecting the exposure of the organism. Extracellular vesicles have been of increasing interest to researchers for over 50 years. The first time EVs were observed and described by transmission electron microscopy in plants (cotton synergids) was in 1965 by William Jensen; he described them as “single-membraned spheres”, associated morphologically with the multivesicular bodies, which were structures believed at that point to be derived from the terminal portion of the endoplasmic reticulum (Jensen, 1965). Two years later, the extracellular vesicles associated with the multivesicular bodies were described by Halperin and Jensen in carrot cell cultures (Halperin and Jensen, 1967; Potestà et al., 2020; Pinedo et al., 2021), and further classified by Marchant et al., in 1968 as para mural-bodies (lonesome and plasmalemmas). Similar structures of small, spherical “particles” were ultra-structurally identified during the same time in Gram-negative bacteria, where Knox et al. (1966) described them as “extracellular globules” (Knox et al., 1966). In mammalian cells, the discovery of the extracellular vesicles is linked with the work of Chargaff and West (1945) on blood clotting, but the first morphological characterization is attributed to Wolf (1967), describing the extracellular vesicles derived from thrombocytes as dud-like expansions and “platelet dust**”** (Wolf, 1967; Couch et al., 2021).

They are applied in many fields as participating elements in pathophysiological processes, including those that favor pathological processes, such as atherosclerosis (Gomez et al., 2020) or thrombosis associated with cancer (Lacroix et al., 2019)**,** and as a reflection of a biological event biomarker. EVs carry a large amount of information due to their lipid, protein, and nucleic acid contents. They were quickly considered potential new biomarkers. They are contained in numerous biological fluids, such as blood, pleural fluid, and urine which is also an advantage in the theatre as a biomarker. Some studies also consider them as therapeutic targets. The EVs derived from plants have been examined for their therapeutic activities and their structure and cargo are similar to that of EVs isolated by mammalian cells. In addition, plant-derived EVs include bioactive lipids, proteins, and mRNAs, and can deliver this important cargo to other cells just like mammalian EVs (Ali et al., 2022). More importantly, they compound several advantageous properties such as antioxidant (Dinicola et al., 2014), antibacterial (Hosseini-Giv et al., 2022), anti-inflammatory (Zhang et al., 2016a), anticancer (Chen et al., 2022), and regenerative potential for various diseases (Zhu and He, 2023).

## 2 Classification of the extracellular vesicle

### 2.1 Extracellular vesicles derived from the mammalian cells

**FIGURE 1:**
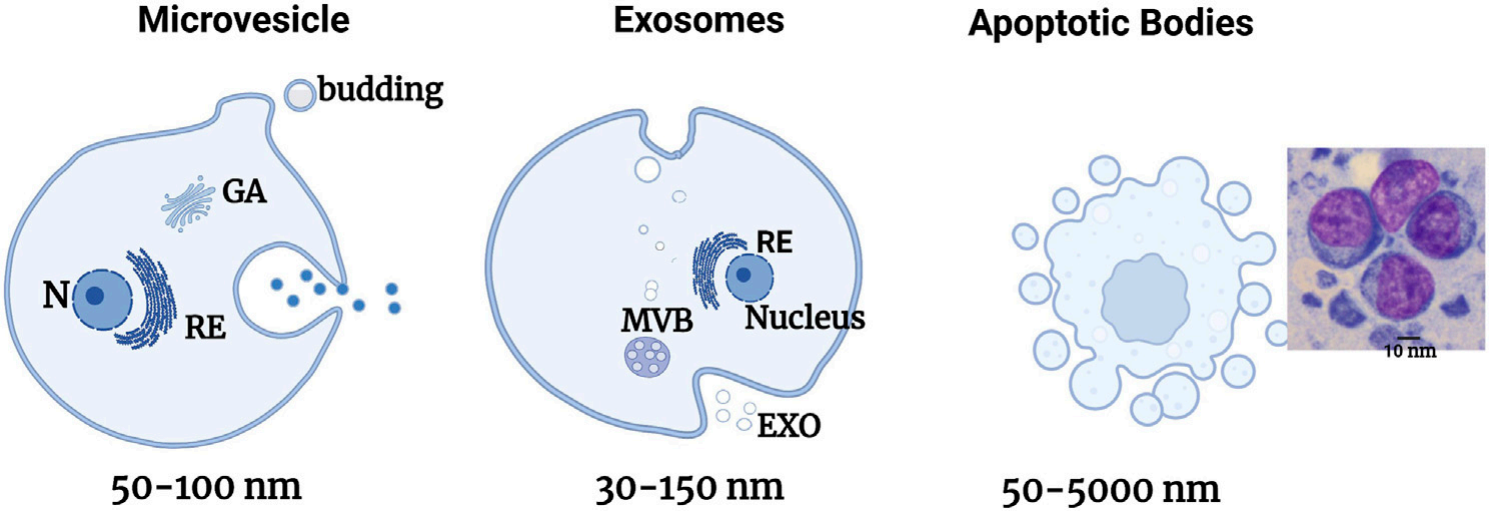
Schematic illustration of the formation of the different EV sub-types. Schematic representation of the liberation of the different types of EVs directly by bourgeoning from the plasma membrane for MPs, by fusing internal multivesicular divisions (MVBs) with the plasma membrane for exosomes, and by bourgeoning from a cell in the process of apoptosis for apoptotic bodies. Adapted from Gurunathan et al. (2019).

Exosomes also called ectosomes, exovesicles, or exosome-like vesicles are uniformly round, membrane-covered structures (He et al., 2021) with a diameter of 30–150 nm (Figure 1) (Tauro et al., 2012)**.** Exosomes are produced by the constitution of multivesicular bodies and secreted to the extracellular milieu after integration into the plasma membrane (Mathivanan et al., 2010; Żmigrodzka et al., 2016)**.** Exosomes are known for their role in intercellular communication and can transport mRNA, bioactive lipids, and proteins (Rayner and Hennessy, 2013; Robbins and Morelli, 2014) and regulate gene/protein expression. Upon contact, exosomes deliver molecules that can confer new characteristics and/or reproduce recipient cells. They are composed of a certain number of specific proteins, notably the tetraspanin family (CD81, CD82, CD9 and CD63, flotillin, TSG101, and Alix) and heat choc proteins (HSP90, HSP70, HSPA5, CCT2, and HSP60) (Théry et al., 2009; AYokoi et al., 2015; Ciardiello et al., 2016; Ha et al., 2016)**.** Exosomal membranes are composed of sphingomyelin, cholesterol, phosphatidylinositol, ceramide, phosphatidylethanolamine, phosphatidylserine, etc. (Tamkovich et al., 2016)**.**

Microvesicles (MV) also called microparticles (MP) are vesicles that bud by burgeoning from the plasma membrane and has a size of 50–1,000 nm or even more (Figure 1) (Gould and Raposo, 2013). MV contains essential protein markers including integrins, selectins, and CD40**.** Their membranes possess cholesterol, diacylglycerol, and phosphatidylserine in greater quantities than exosomes (He et al., 2021)**.** Their constitution is based on its principal mechanisms by a change of membrane phospholipids and the modification of the cytoskeleton of the cell. These MVs then implicate part of the cell’s cytoplasm. They also reflect the state of activation of their cell of origin or its apoptosis (Boireau and Elie-Caille, 2021).

Apoptotic bodies also called apoptosomes are vesicles with the dimension of 1–5 µm delivered by cells in apoptosis (Figure 1) (Zitvogel et al., 1998). They are essentially liberated during cellular apoptosis (Poon et al., 2014). Apoptotic bodies are cleared to assure a “clean” elimination of the cellular content following apoptosis. This content has a strong immunogenic potential, and its release in the extracellular milieu induces local inflammatory reactions. By embedding them in vesicles, the contents of the cell will be eliminated by macrophages that recognize the PS expressed on the apoptotic bodies, thus avoiding any inflammatory responses (Zitvogel et al., 1998). These apoptotic bodies’ function is not limited to the clearance of cellular components since they also participate in intercellular communication.

Extracellular vesicles are distinguished into three groups based on their formation mechanism: exosomes, microvesicles (MV), and apoptotic bodies (AB) (Table 1; Figure 1).

### 2.2 Extracellular vesicle derived from plant cells

Plant-derived VEs are heterogeneous groups of vesicles containing different functions, mainly multivesicular bodies (MVBs), autophagosomes, vacuoles, and exocyst-positive organelles (EXPOs). The size of plant-derived nanovesicles is generally between 50 and 1,000 nm and varies according to plant origin and isolation technique (see Table 2). Plant-derived extracellular vesicles conduct heterogeneous cargos containing different biomolecules (proteins, small RNAs, lipids, and nucleic acids) and are primarily composed of phosphatidic acid, phosphatidylcholine, di galactosyl diacylglycerol, monogalactosyldiacylglycerol, and phytosterols (Liu et al., 2020a; Kocholata et al., 2022). The group of phospholipids occupies several functions including stability, vesicle liberation, and intercellular communication, and participates in the mechanism of membrane fusion**.** In addition, phosphatidylcholine and phosphatidylethanolamine are known for their important roles in strengthening therapeutic activities (antioxidant, antipolitic, and anti-inflammatory) (Wang et al., 2014). The presence of lipid composition in the vesicle membrane has an important role in intercellular interactions and in maintaining vesicle stability under physiological and pathological conditions (Xu et al., 2023). However, small RNAs are present in plant-derived nanovesicles that are capable of regulating biological functions, notably inter-kingdom communication to ensure communication between species as has been indicated in Arabidopsis EVs (Zhang et al., 2016b) in the presence of tiny RNAs (ty RNA) with a length of 10–17 nucleotides, long non-coding RNAs (lncRNA), circular RNAs (circRNA), and small RNAs (sRNA) (Cai et al., 2018). RNAs can be identified in the exterior of EVs, where they can be secured by RNA-binding proteins against enzymatic destruction. In addition, PENETRATION1 was identified between the Golgi complex and the plasma membrane and established as a plant-derived nanovesicle (PDNVs) biomarker (Wang et al., 2010). Syntaxins, which are localized in the membrane of the SNARE family were also established as integrated proteins that are implicated in the transport of vesicles inside the cells (Rutter and Innes, 2017). It has been demonstrated that PENETRATION1 does not localize with ARA6, so it indicates that the biogenic pathway of TET8-positive EVs is not the same as that of PEN1-positive EVs. Certain plant tetraspanins, notably Arabidopsis thaliana TETRASPANIN 8 and TETRASPANIN 9 (AtTET8 and AtTET9) are identified explicitly in infection by the fungal pathogen Botrytis cinerea and colocalize with the Arabidopsis MVB marker Rab5-type GTPase ARA6 inside the cell and EVs at fungal infection sites (Ding et al., 2014). In addition, the Exocyst-positive organelle (EXPO) was recently found to combine with the plasma membrane and release Exo70E2-positive cells in the intercellular spaces during live-cell imaging in plants using the immunostaining technique. The Exo70E2 secretion pathway is free of MVB pathways, and EXPO is unaffected by inhibitors of secretion and endocytosis in protoplasts**.**

**TABLE 2:** Representative plant-derived nanovesicle classification.

Sub-types of Evs	Origin	Size (nm)	Commonly used markers	Ref
Exosomes	Corps multivésiculaires, organites exocystes positifs (EXPO)	<500	-TET8 (tétraspanine 8)	Wang et al. (2010), Rutter and Innes (2017)
			- Pénétration 1 (PEN1	
Microvésicules	Biogenesis unclear	50–1000	-	Caruso and Poon (2018)
Apoptotic bodies	Apoptotic cell plasma membrane blebbing	1000–5000	-	Caruso and Poon (2018)

## 3 Biogenesis of extracellular vesicles

### 3.1 Biogenesis of plant-derived extracellular

**FIGURE 2:**
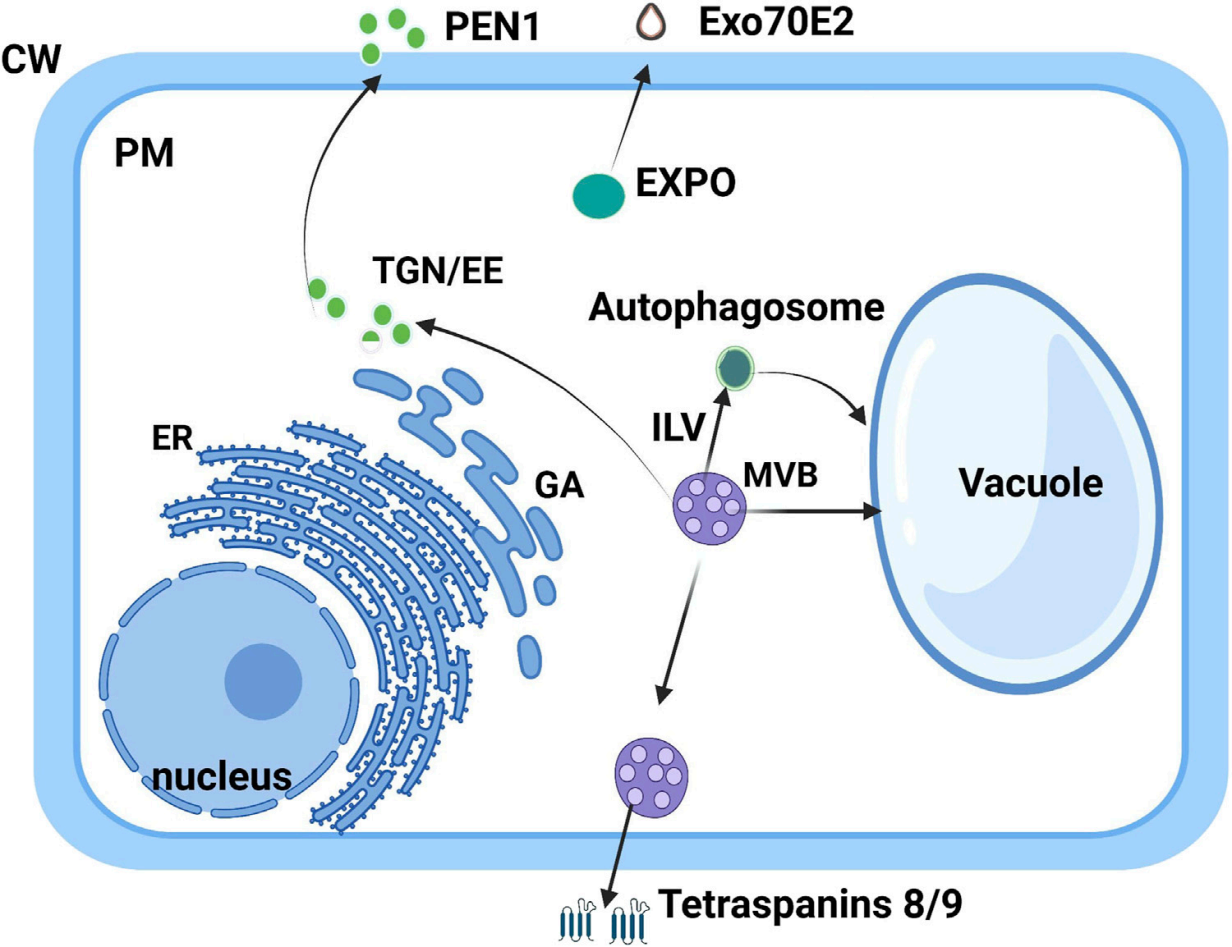
Biogenesis pathway of plant-derived nanovesicles. Adapted from Lian et al. (2022).

Plant-derived nanovesicles generally present as a spherical structure when isolated which could promote cell wall passage**.** Plant-derived nanovesicle biological compounds, which include proteins, small RNAs, and metabolites, are unique in every case and depend on the original cell (Pegtel and Gould, 2019). These two biomarkers PENETRATION1 and TETRASPANIN 8 of EVs, which are not located in the same places are classified in two categories of plant EVs. Tetraspanins occupy a very important role in biogenesis, especially cargo selection, membrane fusion, and exosome absorption (Colombo et al., 2014). Plant-derived nanovesicles’ biogenesis pathway is similar to the exosome biogenesis pathway in that plant nanovesicles associated with TETRASPANIN 8/TETRASPANIN 9 (TET8/TET9) are produced by multivesicular bodies (MVBs). PENETRATION1 is a plant-derived nanovesicle biomarker found in the plasma membrane (Rutter and Innes, 2017). It has been established that trafficking between the Golgi and the plasma membrane is mediated by PEN1. There is no coincidence between PEN1 and ARA6, which determines that the biogenic pathway of TET8 EVs is not the same as that of PEN1 EVs (He et al., 2021). However, another pathway of EV biogenesis has been observed in the EXPO (EXocyst-Positive Organelles), presented generally as a spherical double-membrane structure, compared to the autophagosome, which has been identified in Arabidopsis. Although they present similarities with autophagosomes, which combine with the plasma membrane and liberate membrane vesicles in the cell wall, these vesicles are considered to be exosomes secreted by EXPO (Wang et al., 2010)**.** Indeed, EXPO can combine with Exo70E2 passing the plasma membrane and thus releasing plant-derived nanovesicles (Figure 2). Plant-derived nanovesicles of RNAs are identified in encapsulated EVs, but they are outside the nanovesicles and can be defended by RNA-binding proteins from enzymatic degradation. As has been proved, ty-RNAs of *Arabidopsis thaliana* are abundant in EVs in proportion to cellular RNAs (Baldrich et al., 2019), and only siRNAs of the same RNA precursors can be discovered in EVs. RNA-binding proteins (RBPs) appear to have an important role in loading RNA into EV precursors and in the stability of exotic RNAs. Membrane polysomes could be a site for RNA loading into vesicles. Plant-derived nanovesicles are derived from the endocytic pathway when membranes invaginate during late endocytosis to form intraluminal vesicles (ILVs) inside multivesicular bodies (MVBs) (Zhang et al., 2017). ILVs are generated when premature endosomes integrate with the peripheral membrane layer. They encapsulate plant-derived nanovesicles in MVBs, which combine with the plasma membrane to release exosomes. Although the biogenesis of plant-derived nanovesicles has not been well studied, there is evidence for at least three distinct pathways (Figure 2) (Cai et al., 2019; Nemati et al., 2022)**.**

### 3.2 Biogenesis of mammalian-derived extracellular vesicles

**FIGURE 3:**
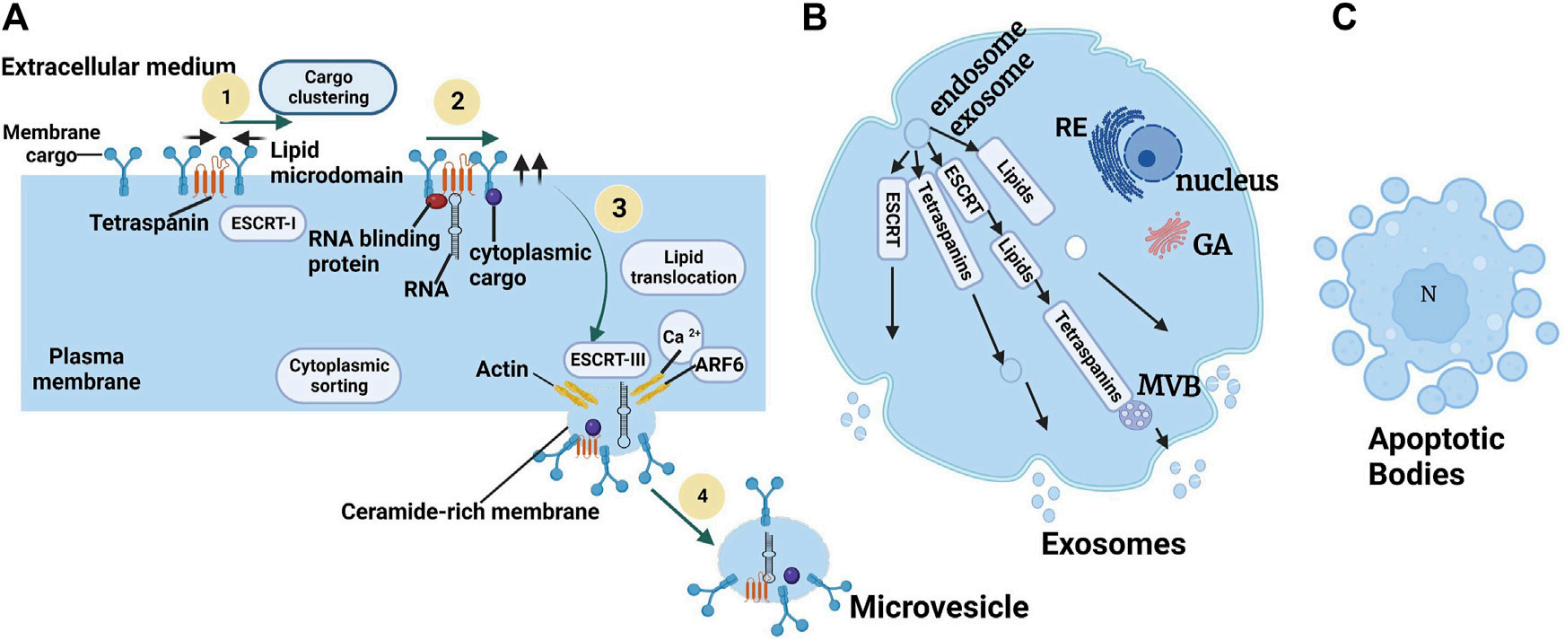
Biogenesis of microvesicles **(A)** ARF6: ADP-Ribosylation Factor 6; ESCRT: Endosomal Sorting Complex Required for Transport (van Niel et al., 2018). **(B)** The molecular machinery of EXO biogenesis: schematization of the different pathways involved in EXO formation involving the ESCRT machinery, tetraspanins, and some lipids. **(C)** Apoptotic body extrusion appears in the scheme and in sections of conventionally embedded apoptotic cells. Adapted from Colombo et al. (2014).

The types of EVs are MVs and EXOs, and CA has various modes of biogenesis. MVs are derived from bourgeoning from the plasma membrane, while EXOs originate in the endosomal system in the form of intraluminal vesicles (ILVs), which are released when MVBs fuse with the plasma membrane, and apoptotic bodies are essentially liberated by bourgeoning from a cell undergoing apoptosis to apoptotic bodies (Poon et al., 2014) (Figure 3). However, even if the formation of MV and EXO occurs at different cell locations, intracellular mechanisms may be implicated in the formation of the two entities, depending on the cell type (van der Pol et al., 2014). According to the discovery in this study (van der Pol et al., 2014), the results indicated that T lymphocytes were capable of forming EVs at the level of the plasma membrane consisting of the characteristics of EXOs. Then, the intracellular mechanisms implied in the two formations simultaneously alter the possibility of establishing different sub-populations of EVs (Colombo et al., 2014).

#### 3.2.1 Biogenesis of exosomes

Exosomes of endosomal origin are confirmed by the analysis of vesicles liberated by immune cells, such as B lymphocytes and dendritic cells (Zitvogel et al., 1998). They are generated by the association of late endosomes with multivesicular bodies (MVBs) which are then released in the extracellular milieu by exocytosis. The constitution of MVBs implies the machinery of the Endosomal Sorting Complex Required for Transport (ESCRT). This machinery is composed of many proteins assembled in four complexes: ESCRT-0, -I, -II, and -III. These complexes have fused proteins such as VSP4, VAT1, ATPase, and TSG101 (Hanson and Cashikar, 2012). In short, before the cascade, we discovered ESCRT-0, which takes care of mobilizing the cargo to the lysosome. It engages ESCRT-I, which makes interaction with ESCRT-II to compose the lipidic vesicle during which the cargos are sequestered. Finally, ESCRT-II engages ESCRT-which takes care of liberating the vesicle in the MVB, acting at the same time with the fifth complex of the machinery: VPS4/VTA1. The formation of EXO independently of the ESCRT complex has been described, implying the synthesis of ceramides. Neutral sphingomyelinase hydrolyzes sphingomyelin to ceramide. This ceramide generates membrane sub-domains that impose a spontaneous negative curvature on the membrane. It has been demonstrated that inhibition of neutral sphingomyelinase 2 (nSMAse-2) prevents ILV bourgeoning in BVMs and EXO liberation by a mechanism independent of the ESCRT machinery (Trajkovic et al., 2008). Exosomes can be detected by different markers, some of which are specific to this type of EV, notably tetraspanin-like membrane proteins (CD9, CD63, CD81, and CD82), or cytosolic proteins involved in endo-lysosomal traffic including Alix, Tsg101, or 14-3-3, major histocompatibility complex (MHC) proteins, heat shock proteins (HSP) chaperones as well as the ESCRT-3 complex binding to the Alix protein (Verderio et al., 2018).

#### 3.2.2 Biogenesis of microvesicles

Knowledge of the biogenesis process of MVs produced from healthy cells is more recent (Minciacchi et al., 2015). The biogenesis of MVs results from a remodeling of the cytoskeleton induced by an increase in intracytosolic Ca2+ concentration (Al-Nedawi et al., 2008) (Figure 3A). The increase in Ca2+ stimulates enzymatic machinery providing membrane phospholipid asymmetry, including aminophospholipid translocases (flippases and floppies), scramblases, and calpain. The activation of the enzymatic machinery leads to the externalization of phosphatidylserine (PS) from the inner to the outer leaflet of the plasma membrane. The loss of membrane asymmetry induces an excess of negative charge at the surface of the plastic membrane (Piccin et al., 2007). This excess charge is responsible for membrane curvature and cytoskeletal reorganization favoring MV release (Jimenez et al., 2003; Del Conde et al., 2005). Inhibition of scramblase has been shown to suppress PS externalization in platelets and the formation of procoagulant MVs (Jimenez et al., 2003)**.** However, even when membrane phospholipid asymmetry is maintained, MVs can be formed (Del Conde et al., 2005)**.** These observations indicate that other lipids, and the domains they structure, contribute to the biogenesis of MVs. An important membrane lipid component is cholesterol, which is present in MVs. Depletion of membrane cholesterol has been shown to decrease MV production by THP-1 monocytes (Li et al., 2012). In addition to membrane remodeling and cytoskeletal rearrangement, other regulators are necessary for MV biogenesis. The activity of small GTPases of the RHO family and ROCK (RHO-associated protein kinase), which are important regulators of actin dynamics, induces MV formation in different tumor cells**.** (McConnell et al., 2009).

Release of MVs requires rupture of the plasma membrane. This mechanism depends on the interaction of actin and myosin with ATP-dependent contraction. (Muralidharan-Chari et al., 2009)**.** In cancer cells, it has been shown that activation of ARF6 (ADP-Ribosylation Factor 6), ADP Ribosylation Factor 1 (ARF1), and small GTP-binding proteins lead to phosphorylation of the myosin side chain of myosin and contraction of actomyosin, permitting MV to detach from the membrane (Nabhan et al., 2012). Another regulator of actin dynamics, the Cdc42 (Cell division control protein 42 homolog), is involved in MV release in HeLa cells; however, the mechanism is unknown (McConnell et al., 2009)**.** In another study, TSG101 (Tumor Susceptibility Gene 101 Protein) and ATPase VPS4, which are mainly involved in the EXO formation via the ESCRT machinery, were reported to be involved in the fission and release of MV (Wehman et al., 2011)**.** The involvement of the ESCRT machinery in MV release has also been demonstrated in *C. elegans* embryos (Bianco et al., 2005)**.** Another possible pathway for MV release is the activation of the ATP-dependent P2X receptor 7 (P2X purinoceptor 7), which is ATP-dependent. Activation of the receptor results in membrane rearrangement that influences MV release (Bianco et al., 2009)**.** This process is associated with the translocation of acid sphingomyelinase to the plasma membrane, which produces ceramide, favoring membrane bourgeoning and MV liberation. (Reátegui et al., 2018)**.**

#### 3.2.3 Biogenesis of apoptotic bodies

Apoptotic bodies are larger than other vesicles and carry particular markers (thrombospondin, complement component 3, and C3B) (Akers et al., 2013; Duijvesz et al., 2015)**.** Apoptotic bodies produce apoptosis-induced cell death (Suárez et al., 2017)**.** They are fragments of the cell formed by blebbing. This phenomenon is produced when ruptures are formed in the actin cortex caused by actomyosin contractions. This creates weak points in this actin cortex. These weak points end up inflating under the hydrostatic pressure of the cell providing bourgeoning called blebs. The actin is then rearranged in these blebs, leading to membrane scission (Suárez et al., 2017)**.** The vesicles are thus liberated into the extracellular milieu (Akers et al., 2013; Suárez et al., 2017)**.** Some smaller vesicles can also be produced during apoptosis (apoptotic vesicles), but the mechanisms that control their production and release are not yet clearly defined (Suárez et al., 2017).

## 4 Different methods of isolating extracellular vesicles

Extracellular vesicles are generally isolated from different biofluids. Given their generosity in terms of size and concentration, and depending on their mode of biogenesis and cellular origin, the separation of these extracellular vesicles is according to their sub-types and sub-populations of EVs, using different isolation techniques or methods (Boireau and Elie-Caille, 2021).

**FIGURE 4:**
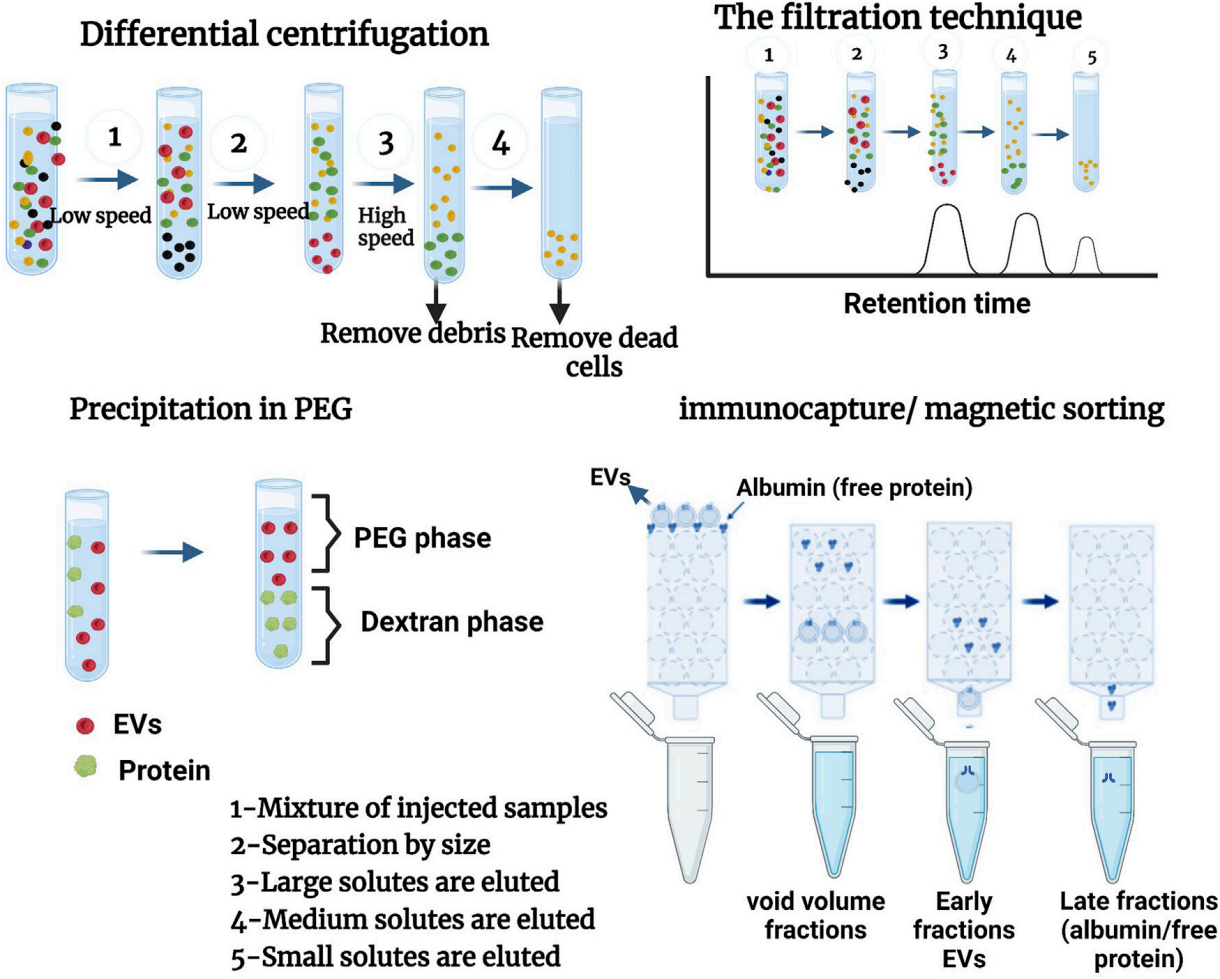
different methods of isolation of EVs. Adapted from Monguió-Tortajada et al. (2019), Boireau and Elie-Caille (2021), Chhoy et al. (2021).

Nowadays, several methods of EV purification exist, but not all of them allow for obtaining the same degree of purification and the same level of concentration of the sample. For this reason, some techniques are called “concentration” techniques, such as differential centrifugation, which allows only a slight purification of the sample even when several rounds of washing are carried out, whereas other techniques are called “purification” techniques because they allow a real separation between the EVs and the soluble proteins of the surrounding medium. At present, many teams are proposing a combination of several methods for purifying EVs. This section will examine the different reference techniques used to isolate EVs as indicated in Figure 4.

### 4.1 Ultracentrifugation

Ultracentrifugation is the most frequently used method for isolating EVs. This method has been used for studying EVs derived from cell culture supernatants and biological fluids (Campoy et al., 2016; Coughlan et al., 2020)**.** Some advantages of ultracentrifugation are that it can isolate EVs from large volumes of biological fluids, requires a relatively restricted set of reactants and consumables, and has no impact on EVs apart from gravitational force and pipetting (as no chemicals that are likely to interfere with downstream EV analysis are used). To reduce debris caused by co-sedimentation and contamination of preparations by cell lysis products, this phase also includes several sub-phases, firstly by centrifugation at 300–400 × g for 10 min, secondly sedimentation of a large proportion of the cells at 2000 × g to remove cell debris, thirdly at 10,000 × g to eliminate biopolymer aggregates and apoptotic bodies, and finally to obtain contiguous EVs in the supernatant, which are increased by ultracentrifugation at (100,000–200,000 × g) over 2 h.

#### 4.1.1 Ultracentrifugation differential

Differential ultracentrifugation (UC) is a well-known method for isolating EVs in favor of its ease of use and its low character accessibility (Witwer et al., 2013; Pérez-Bermúdez et al., 2017). It is used for separating large EVs (by centrifugation at around 10,000 g) and small EVs (ultracentrifugation at 100,000 g). Differential ultracentrifugation pellets large extracellular vesicles at a slow centrifugation speed and small ones at a high speed (100 000 g). Macromolecules and/or lipoproteins can be co-precipitated with the extracellular vesicles (Blandin and Le Lay, 2020).

#### 4.1.2 Density gradient ultracentrifugation

Density gradient ultracentrifugation is a classic technique for isolating groups of vesicles based on their density and buoyancy velocity (Cai et al., 2018). This enables them to improve the purification of extracellular vesicles. The flotation of the isolated EVs on a density gradient permits the separation of macromolecular complexes and/or lipoproteins co-precipitated with the EVs. In addition, this method permits excellent results in terms of EV fraction purity and the number of EV proteins and RNA to classical ultracentrifugation and commercial kits (Van Deun et al., 2014). Moreover, it is established that EV preparations isolated from this solution are deprived of microvesicles superior to 200 nm in contrast to EV obtained by other methods (Lobb et al., 2015). At present, density gradient ultracentrifugation is frequently used to isolate microvesicles. However, this method leads to a decrease in the size of EVs; it is complex, tedious, time-consuming (up to 2 days), and requires expensive materials (Lobb et al., 2015; Zeringer et al., 2015).

### 4.2 The filtration techniques

Diverse techniques have been invented to filter EVs. These include cross-flow filtration (CFT) and centrifugal ultrafiltration (UF). In the last stage, high pressure is used to increase the density of smaller particles, permitting them to pass through the membrane without being blocked by clumps. In addition, UF provides a cut-off point for damage to its membranes due to the weight of the particles. This permits the removal of any contaminants found in extracellular vesicles using TFF. Sequential UF, consisting of a succession of filtration steps with a gradual reduction of the cut-off point, can permit EV isolation and enrichment from complex biological fluids (Boireau and Elie-Caille, 2021).

#### 4.2.1 Gel filtration (size exclusion chromatography)

Size exclusion chromatography is a classical technique for the fractionation of biological entities that establish isolation in proportion to the dimension of the object or its hydrodynamic volume (Boireau and Elie-Caille, 2021). It is used as a porous matrix where EVs smaller than the pore size will be reserved for a long time according to their elution of more advanced large-sized EVs (Blandin and Le Lay, 2020). It is largely applied to the preparation of biopolymers (proteins, polysaccharides, proteoglycans, etc.). As demonstrated, this technique can also be used to separate EVs from protein and lipoprotein complexes in blood plasma and urine (Böing et al., 2014; Muller et al., 2014; Lozano-Ramos et al., 2015; Gámez-Valero et al., 2016), which is difficult, and many other methods have failed (Mathivanan et al., 2012). Gel chromatography is an efficient and rapid technique permitting the isolation of EVs efficiently without loss of high reproducibility. In particular, exosomes have a very large hydrodynamic radius in comparison with proteins, lipoproteins, and protein complexes, and they can be separated very well from these components. However, the size of some of the chylomicrons is similar to that of the isolated vesicles; similarly, EV preparations made in this manner compound lipoproteins but at a much slower rate than that found in the case of other techniques applied to EV isolation (Yamamoto et al., 1970).

### 4.3 Immunocapture or magnetic sorting illustrated

Immunocapture or magnetic sorting is a technique that permits the retention of EVs expressing a surface antigen (e.g., CD9, CD63, or CD81) using monoclonal antibodies (directed against this antigen) coupled to magnetic beads retained on a column by magnets. In the absence of a magnetic field, EVs positive with this antigen are then eluted. The isolation of sub-populations of EVs can also be performed by immuno-capture using antibodies specifically directed against antigens carried by EVs, such as tetraspanins (Blandin and Le Lay, 2020). This technique consists of isolating a particular sub-category of EVs from balls coated with an antibody that recognizes the specific protein marker exposed on EV membranes. It can also prevent contamination of isolated EVs by cytoplasmic proteins or RNA (Van Deun et al., 2014).

### 4.4 Precipitation in PEG

This is a method that uses polymer solutions with dextran to induce phase separation or extracts that will induce the precipitation (at low-speed centrifugation) of VEs by its surfactant properties, and polyethylene glycol (PEG) retains the macromolecules and other molecular components. The precipitation of EVs by polymer mixtures (Dextran, polyethylene glycol, or PEG) permitting phase separations at a low centrifugation speed and the precipitation of EVs is the basic principle of many commercial kits (e.g., Exo Quick TCTM). These last kits present the advantage of being rapid and easy to use but often lead to the co-precipitation of macromolecular protein complexes, lipoproteins, or immunoglobulins (Blandin and Le Lay, 2020). PEGs of diverse molecular weights have been used for many years for the precipitation of proteins, nucleic acids, viruses, and other small particles (Yamamoto et al., 1970). The technique uses a diminution of and the solvency of elements in super hydrophilic polymer solutions, PEG. The procedure is limited to the combination of polymer solution and sample, incubating and pelleting the EVs by low-speed centrifugation (1,500 × g).

In conclusion, there are other methods for isolating extracellular vesicles, such as microfluidic systems, immunological separation, and microfluidics. This number continues to grow in parallel with technological advances.

### 4.5 Isolation of mammalian-derived extracellular vesicles

At this moment, various methods have been used to isolate mammalian-derived EVs, primarily ultracentrifugation, differential centrifugation, density gradient, and size exclusion chromatography. However, not all these methods are adapted to the evolution of extracellular vesicle production. These methods require several complicated and repetitive centrifugation steps to remove all debris. In addition, these steps are not yet well-detailed in the literature. This observation has permitted some researchers to develop new methods to purify EVs using simple procedures such as those mentioned in the following studies such as isolation of EVs derived from human milk by ultracentrifugation and filtration. For example, separate milk samples by different centrifugations (6,500 × g at 4°C for 30 min and 12,000 × g at 4°C for 1 h) were used to remove debris. Next, skimming was affected using 0.45 and 0.22 μm filters to remove any remaining debris. The filtered supernatant was centrifuged at 135,000× g for 90 min at 4°C to granulate the exosomes (Reif et al., 2020; You et al., 2021). In another study, EVs derived from milk were isolated by differential centrifugation; for density gradient, the milk was centrifuged twice at 3,000 × g, and then the milk supernatant was subjected to differential centrifugation at 5,000 × g and 10,000 × g in new, sterilized SW40 tubes. The supernatant at 10,000 × g was again added to a sucrose gradient (ranging from 2.0 to 0.4 M sucrose) and centrifuged at 192,000 × g for 15–18 h. At the final stage, the samples were collected, combined, and centrifuged at 100,000 × g for 65 min, the supernatant removed, and the EV pellets aliquoted and stored at 80°C (Van Herwijnen et al., 2018a). Bovine milk was isolated by the acetic acid/ultracentrifugation (AA/UC) method. The first skimmed milk was heated for 10 min at 37°C, then mixed with acetic acid, followed by centrifugation at 10,000 g for 10 min at 4°C. The supernatant was filtered through a 0.22 μm membrane and designated as lacto-serum. Lacto-serum was ultracentrifuged at 210,000 g for 70 min at 4°C. An EV pellet was resuspended with phosphate-buffered saline (PBS) for clean-up, and the remaining precipitates were removed by centrifugation at 10,000 g for 5 min at 4° (András and Toborek, 2015). In Table 3 we have summarized several studies on the Isolation of mammalian-derived extracellular vesicles.

**TABLE 3:** Summary of isolation methods of mammalian-derived extracellular vesicles.

Derived mammalian	Isolation methods	Product	Ref
Cow milk/solid tumors	differential centrifugation	100,000 g during 1 h at 4°C derived from cow milk	Van Herwijnen et al. (2018b)
Cow milk EVs	differential centrifugation; density gradient	100,000 g during 65 min at 80°C	Munagala et al. (2016)
Bovine breast milk-derived EVs	-Acetic acid/ultracentrifugation (AA/UC) method	−10,000 g during r 5 min at 4°C	Somiya et al. (2018)
	Centrifugation/ultracentrifugation (C/UC) method	- 130,000 g during 60 min at 4°C	
Porcine milk	Centrifugation	2000 g for 30 min at 4°C	Dignat-George and Boulanger (2011)
osteoblasts-derived EVs	Centrifugation	300 × g during r 90 min at 25°C	O'Brien et al. (2020)
Oviduct derived EVs	Centrifugation	100,000 g during 70 min at 4°C	Fu et al. (2020)

### 4.6 Isolation of plant-derived extracellular vesicles

**FIGURE 5:**
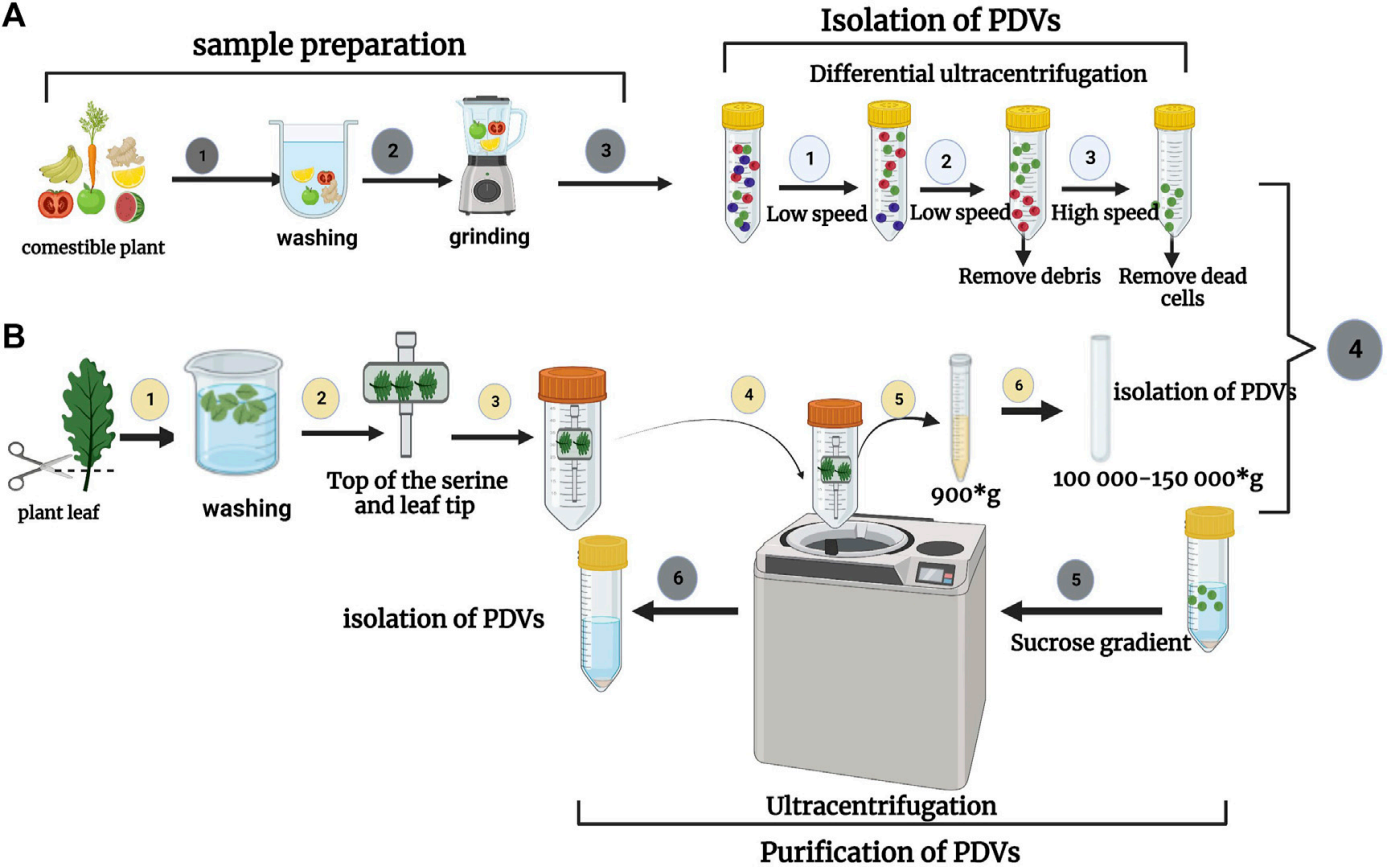
**(A)** Isolation process of nanovesicles derived from a comestible plant (fruit or vegetable) as a first step of sample preparation (mixing, pressing, or grinding). The second step is the isolation and purification of plant-derived nanovesicles (e.g., by differential ultracentrifugation and sucrose gradient ultracentrifugation). **(B)** The process of isolating EVs from plant leaves. Initial steps to isolate the fluid of apoplastic wash from detached plant leaves. The leaves are then cut using a pair of scissors. The leaves are put into a syringe, then deposited on a piece of transparent adhesive tape and rolled onto the syringe. The taped leaves are then placed in a 50 mL conical tube and collected by centrifugation. Adapted from Li et al. (2023a).

There are several methods for the isolation of plant-derived nanovesicles (PDNVs), for example, differential ultracentrifugation (UC) combined with sucrose density gradient centrifugation, PEG precipitation, size exclusion chromatography (SEC), ultra-filtration membrane separation, etc., Nevertheless, in practical terms (extraction rate, extraction time, and purity) none of these methods are excellent, but the combination of several methods seems to be the best possible solution. The advantages and disadvantages of these techniques are established. Differential ultracentrifugation and the extraction method are the most used techniques for isolating nanovesicles of plant origin, then they are purified by density centrifugation on a sucrose gradient. Different types of nanovesicles can be selected based on centrifugation density and sucrose gradient (Yang et al., 2018a). The main stage of the UC step is to prepare the sample including mixing, pressing, or grinding, then plant-derived nanovesicles (by differential ultracentrifugation and sucrose gradient ultracentrifugation) are isolated and purified. In a centrifuge, the supernatant is initially centrifuged at a low speed to remove fiber debris in the plant tissue; in a second step, the centrifugation speed is gradually increased to remove finer particles while preserving plant-derived EVs in the supernatant. Evidently, as the number of centrifugations increases, the speed of centrifugation gradually increases and the duration of centrifugation becomes longer and longer. After three centrifugations at a relatively low speed, the supernatant is maintained. At this point, a centrifugal force of between 100,000 and 150,000 g is selected for the second centrifugation. (Li et al., 2023a). (See Figure 5). In Table 4 we have summarized several studies on the Isolation of Plant-derived extracellular vesicles.

**TABLE 4:** Summary of isolation methods of plant-derived extracellular vesicles.

Derived-plant	Isolation methods	Yield	Ref
Carrot	UF/SEC	3, 2 ×^1011^ particles/g carrot	Kim and Rhee (2021)
Ginseng	DUC/GUC	∼ 500 mg/kg ginseng	Cao et al. (2019)
Tomato	DUC/GUC	3,8 × 1,016 particules/kg tomato 26 ± 11 mg/kg tomato	Bokka et al. (2020)
Orange Juice	DUC/SEC	2,6 × 1,015 particules/kg tomato 6,9 ± 1.5 mg/kg tomato	Berger et al. (2020)
Cabbage	UF/SEC	1,504 ×^1011^ particles/mL	You et al. (2021)
*endropanax morbifera sap*	UF	∼ 10 mg protein/10 g sap	Kim et al. (2020)
*Arabidopsis thaliana*	DUC/GUC	7,2 × 1,010 particles/g leaf	Liu et al. (2020b)
Ginger	DUC/GUC	4,2 × 109 particles/g Ginger	Teng et al. (2018)
Ginger	DUC	0,72 × 1,010 particules/mL jus 1.78 μg protein/mL juice	Li et al. (2018)
Ginger	DUC	0,5 à 2 × 1,011 vesicles/g ginger	Chen et al. (2019)
Strawberry	DUC	18 ± 3 μg/250 mL juice	Perut et al. (2021)
Grapefruit	DUC	5,7 ± 0,7 × 1,013 particles/mL	Garaeva et al. (2021)
Ginger	Polymer-based precipitation	∼20 g/kg ginger (at pH 4) ∼4 g/kg ginger (at pH 7)	Suresh et al. (2021)
*Dendropanax morbifera*	UF	1.53 × 109 particles/g (from leaf) 4.98 × 108 particles/g (from stem)	Lee et al. (2019)

## 5 Therapeutic effects and application of extracellular vesicle

### 5.1 Therapeutic effects and application of EV derived from plants

The biological activities of exosome-type nanovesicles, in their intact natural structural integration with their bioactive cargoes after their simple isolation from plant EVs, diminish pathological situations in species of other reigns and provide multiple therapeutic alternatives (Mu et al., 2014; Dad et al., 2021).

To give an overview of the potential therapeutic application of plant-derived extracellular vesicles (P -EVs) based on plant types, plant comestibles have in recent years been the focus of numerous studies, which have revealed encouraging specificities that explain their availability, biocompatibility, and biodegradability. These EVs could well be necessary for the therapy of many diseases. (Kameli et al., 2021).

More importantly, they consist of several advantageous properties such as Ginger which has various beneficial properties including anti-oxidant (Hung et al., 2017)**,** antibacterial (Dinicola et al., 2014), anti-inflammatory (Rome, 2019), anti-cancer (Vislocky and Fernandez, 2010), and regenerative potential for various diseases (Brahmbhatt et al., 2013). In addition, plant-derived nanovesicles are continents of biomolecules including proteins, drugs, DNA vectors, and siRNAs, and are delivered to target tissues (Nemati et al., 2022). It is for this cause that the biomolecules of plants have gotten much attention from researchers for their potential to improve health and defend against diverse infections.

In recent years, in the field of nanotechnology, nanovesicles derived from edible plants have attracted the attention of scientists for their drug delivery potential, as these particles can deliver hydrophobic and hydrophilic therapeutic agents to disease target sites. We will describe studies that have proven the therapeutic efficacy of nanovesicles derived from edible plants (grape, grapefruit, ginger, lemon, and carrot).

Ginger has intrinsic chemical compositions, such as shoal and gingerol, possessing several health benefits. In addition, multiple studies have indicated the therapeutic effects of ginger, including these studies (Zhang et al., 2016b; Zhu and He, 2023) where the authors have studied the effects of ginger in regeneration. Ginger-derived nanovesicles have demonstrated their role in reducing the expression of secreted hemotoxic protein and influencing the expression of other mitochondrial and cytoplasmic proteins such as heat shock protein, axing, and kinesin for intestinal wound recovery (Kim et al., 2022). Further studies have been interested in the effect of ginger in antitumor activities; the research found that plant-derived miRNAs are capable of crossing species and performing a veritable regulatory role in the human organism (Liu et al., 2017)**.** Furthermore, ginger-derived nanovesicles have been demonstrated to reduce cyclin D1 RNA levels in mice with colorectal cancer (Zhang et al., 2016b)**.** Studies indicate that ginger has hepatoprotective properties against ethanol- and acetaminophen-induced hepatotoxicity of carbon tetrachloride (Zhuang et al., 2015).

Grapefruit-derived nano-vectors (GNVs) presented varieties of therapeutic agents, including chemotherapeutics, DNA expression vectors, siRNAs, and proteins such as antibodies. It has been reported that particles purified from grapefruit indicated high stability and can carry several agents such as curcumin and Zymosan that were functionally active. Grapefruit-derived nanovesicles had similarities in size and structure to mammalian-derived exosomes. They consisted of proteins, lipids, and miRNA and have been assimilated by intestinal macrophages and stem cells. In another study, Wang et al. coated grapefruit-derived nanoparticles (GDNV) rich in active anti-inflammatory leukocyte receptors (IGNV). Using various animal models of inflammation-induced diseases, they have demonstrated that IGNVs target inflamed tumor tissue better than GDNVs. Moreover, this targeting of inflamed tissue was significantly inhibited by blocking LFA-1 or CXCR1/CXCR2 on IGNV membranes. Wang et al. reported that grapefruit-derived nanovesicles could release chemotherapeutic agents, siRNAs, DNA expression vectors, and proteins in different cell types. They also simultaneously administered grapefruit-derived nanovesicles and folic acid, and reported that this strategy significantly increased the efficiency of targeting cells expressing folic acid receptors. They then demonstrated that these nanovesicles improved chemotherapy-induced tumor growth inhibition in CT26 and SW620 cell-derived tumors in mice (Wang et al., 2015).

Nanovesicles derived from Citrus limon (lemon) contain many benefits, including antibacterial, antifungal, anti-inflammatory, anticancer, hepato-regenerative, and cardioprotective activities (Bhavsar et al., 2007; Kim et al., 2012; Riaz et al., 2014; Parhiz et al., 2015; Otang and Afolayan, 2016)**.** The pharmacological potential of citrus lemon is determined by its rich chemical composition such as phenolic acids, coumarins, carboxylic acids, amino acids, and vitamins. In addition, some studies have demonstrated that citrus lemon nanovesicles inhibited the growth of chronic myeloid leukemia (CML) tumors *in vivo* by specifically reaching tumor sites and activating the TRAIL-mediated apoptosis process. Also, they were studied for their role in reducing tumor growth. They were capable of curbing *in vivo* tumor development in chronic myeloid leukemia (CML) by targeting tumors, reducing oxidation, and reducing cancer risk (Raimondo et al., 2015). In another study, citrus limon-derived nanovesicles were shown to possess cell growth inhibitory effects, primarily in p53-inactivated CRC cell lines, via the macropinocytosis pathway. The results of this study indicate that p53 inactivation activated macropinocytosis activity and that citrus lemon-derived nanovesicles had a cell growth inhibitory effect via the macropinocytosis pathway (Takakura et al., 2022).

Extracellular vesicles derived from carrot contains phytochemicals, namely, phenolics, carotenoids, polyacetylenes, and ascorbic acid. These chemical products help reduce the risk of cancer and cardiovascular disease because of their antioxidant, anti-inflammatory, plasma lipid-modifying, and anti-tumor properties. The role of polyphenols in the prevention of degenerative diseases, such as cancer, cardiovascular diseases, and neurodegenerative diseases has been reported. Carrot (Daucus carota) is also used in research for its medical effects.

Carrot juice also contains glutathione, an antioxidant that protects against free radicals. It presents potent anti-inflammatory properties that permit the relief of rheumatic and arthritic symptoms (Metzger et al., 2008). A study establishing that carrot-derived nanovesicles have anti-inflammatory and antioxidant effects that can restore glucose tolerance and cardiovascular and liver functions has been assessed in an *in vivo* model (Poudyal et al., 2010). In another study, EVs derived from carrots (Carex) were studied as a novel biomaterial with antioxidant functions in cardiomyoblastoma and neuroblastoma cells. The results indicated similar properties to EVs, and their antioxidant and apoptotic effects in cardio-myoblasts and neuroblastoma cells were further studied. Carex significantly inhibited ROS production and apoptosis induction; therefore, the antioxidant effect of Carex may be more effective in the early phase of diseases. Carex presented low cytotoxicity in H9C2 cardiomyocytes and SH-SY5Y neuroblastoma cells when high levels of Carex were delivered to cells. In addition, Carex inhibited the reduced expression of antioxidant molecules, including Nrf-2, HO-1, and NQO-1, in both models (Kim and Rhee, 2021).

Grape (Vitis vinifera) nanovesicles and their bioactive compounds have several pharmacological activities such as antioxidation and risk reduction and contain several active components including flavonoids, polyphenols, anthocyanins, proanthocyanidins, procyanidins, and resveratrol, a derivative of stilbene. It has a wide range of pharmacological and therapeutic effects, such as antioxidant, anti-inflammatory, and antimicrobial activities, as well as cardioprotective, hepatoprotective, and neuroprotective effects.

Wang et al. (2013); Zhuang et al., 2016 reported that grape-derived nanovesicles contain microRNA, proteins, and lipids. Although these nanovesicles do not resemble mammalian cell-derived exosomes, their structure and composition are similar to mammalian cell-derived exosomes (Ju et al., 2013). Grape-derived nanovesicles consist of proteins such as aquaporins and HSP70 proteins enriched in phosphatidylethanolamine. A study has indicated that grape-derived nanovesicles present unique transport characteristics and biological functions (Ju et al., 2013; Yu et al., 2020). These nanovesicles can traverse the intestinal mucosal barrier and be taken up by mouse intestinal stem cells, significantly inducing intestinal stem cells through the Wnt/β-catenin pathway (Yu et al., 2020). In addition, nanovesicle grape derivatives can reduce many of the risk factors associated with cancer, cardiovascular health, age-related cognitive diseases, and neurodegenerative diseases. These effects are generally attributed to the function of flavonoids in grapes, antioxidant activity, and increased nitric oxide production (Vislocky and Fernandez, 2010).

In terms of therapeutic applications, the extracellular vesicles released by plant cells possess several therapeutic agents, including drugs, proteins, DNA vectors, and siRNAs, and deliver them to target tissues, making them promising natural resources for modern drug discovery. In addition, they also reduce the risk of various pathologies as mentioned above, including cancer, chronic inflammatory diseases, and others. Because of their drug delivery potential, as these particles can deliver both hydrophobic and hydrophilic therapeutic agents to targeted disease sites, there has been a lot of attention from scientists which shows that extracellular vesicles are positioned as an ideal candidate for therapeutic applications. However, plant-derived nanovesicles can locate intrinsically in target tissues, which is one of the most important characteristics of a targeted delivery system. All aspects of plant-derived nanovesicles have not yet been fully identified and described, as they represent a new concept in the field of nanomedicine. The therapeutic potential of plant-derived nanovesicles has recently been demonstrated in several disease models and has been widely applied in the development of new drugs to treat particular diseases or maintain healthy body functions. A variety of plants have been used for the isolation of therapeutically effective exosomes presenting diverse functionalities. Table 5 lists important plants that have been used to extract plant-derived nanovesicles and their therapeutic applications.

**TABLE 5:** Therapeutic application of plant-derived nanovesicles.

Plant-derived nanovesicles source	Therapeutic application	Ref
Arabidopsis (*Arabidopsis thaliana*)	Arabidopsis EV secretion is enhanced under biotic stress, and the EV proteome was modified in response to P. syringe infection	Liu et al. (2020a)
Cucumber (*Cucumis sativus*)	Description of cucumber-derived exosome vesicles	Abraham et al. (2022)
Aloe vera (*Aloe vera barbadensis*)	The vesicles are absorbed by macrophages marrow-derived macrophages and prevent activation of the NLRP3 inflammasome	Budai et al. (2013)
Broccoli (*Brassica oleracea*)	Broccoli-derived nanovesicles have beneficial effects in the inhibition of colitis in treated mice	Deng et al. (2017)
Coconut (*Cocos nucifera*)	Characterization of small RNAs in nanovesicles; miRNAs regulate the expression of inflammatory cytokines and cancer-related genes *in vitro*	Xiao et al. (2018)
Apple (*Malus domestica*)	Apple-derived nanovesicles have a powerful anti-inflammatory effect *in vitro*	Trentini et al. (2022)

In conclusion, to advance therapeutically, drug nanocarriers require a thorough evaluation of their physicochemical characteristics and communications in various biological environments (Herrmann et al., 2021). While liposomes have been largely evaluated, EVs have indicated unique properties that make them superior for drug delivery systems (Kooijmans et al., 2016). Plant-derived nanovesicles can be used as drug carriers as they present advantageous properties including low immunity, tissue-specific targeting, safety, large-scale production, preferred negative zeta potential values, and the ability to load many biomolecules. In Table 5, we present a summary of another plant-derived nanovesicle for therapeutic use.

### 5.2 Therapeutic effects and application of mammalian-derived extracellular vesicles

We will discuss different studies that attribute various therapeutic functions to mammalian-derived extracellular vesicles, e.g., extracellular vesicles of an endosomal origin that are released by different cell types (neurons) serve to remove unwanted proteins in a drainage system and also serve to intercellularly transport their cargo: a specific set of proteins, RNAs, and lipids. Recently, extracellular vesicles derived from mouse neuroblastomas captured Aβ, resulting in reduced amounts of Aβ, decreased amyloid deposition, and reduced Aβ-induced synaptotoxicity in the hippocampus. This demonstrates the role of neuroblastoma cell extracellular vesicles in Aβ release (Yuyama et al., 2014). Results in another study were also similarly described on neuron-derived EVs (Yuyama et al., 2015). On the other hand, endothelial extracellular vesicles secreted by activated or apoptotic endothelial cells may be capable of participating in both toxic and positive effects of the vascular endothelial response. These effects may include anticoagulation, anti-inflammatory effects, angiogenesis, endothelial survivance, and endothelial regeneration (Dignat-George and Boulanger, 2011). In another study of miRNAs presented in human and porcine milk, EVs focused on a variety of genes that were involved in the regulation of the epithelial barrier and neonatal defense; these miRNAs associated with breast milk EV contributed to the subsequent guided development of the newborn (Van Herwijnen et al., 2018b). However, another study indicated that extracellular vesicles in pig milk also attenuated deoxynivalenol-induced changes in body weight and intestinal epithelial growth in mice. These extracellular vesicles inhibited cell proliferation and the creation of tight junction proteins and reduced deoxynivalenol-induced apoptosis. EVs also increased the expression of miR-181a, miR-30c, miR-365-5p, and miR-769-3p in IPEC-J2 cells, then reduced the expression of their target genes in the p53 pathway, and finally attenuated DON expression by promoting cell proliferation and tight junctions and inhibiting apoptosis to induce lesion (Xie et al., 2020)**.** In addition, a study of cow’s milk-derived extracellular vesicles that reduced primary tumor growth and attenuated the progression of weight loss problems in cancer-related body weight loss was examined using the C-26 colorectal tumor model. Their discoveries highlighted the role of milk-derived extracellular vesicles in interspecies communication and their considerable context-dependent role in regulating cancer progression and metastasis. However, their results also suggested that milk-derived extracellular vesicles possess antiproliferative properties on cancer cells *in vitro* (Zhuang et al., 2016), as demonstrated in similar studies (Munagala et al., 2016). It has been indicated that peripheral endothelial cell-derived EVs may be involved in the modulation of innate immunity (Palette et al., 2013). In addition, human dermal microvascular endothelial cells have selectively sequestered cytoplasmic RNA degradation mechanisms in exosomes, which could also be involved in gene regulation**.** It has been established that dendritic cell-derived EVs can be focused to deliver siRNAs to neurons, microglia, and oligodendrocytes in the mouse brain (András and Toborek, 2015; Somiya et al., 2018). In another study, EVs derived from umbilical cord polyvalent mesenchymal stromal cells presented anti-apoptotic, pro-angiogenic, and antifibrotic activities, and immunomodulatory effects; effects similar to those of their source cells (Rohde et al., 2019).

In terms of therapeutic application, after confronting a series of challenges when developing a therapeutic approach including toxicity, safety, target specificity and large-scale production, leading to several targeting strategies for drug delivery in preclinical and clinical settings. Mammalian-derived nanovesicles are promising for clinical applications, both as biomarkers and as therapeutic vectors. As proven by scientists, EVs can be used as therapeutic agents or as drug cargo for reaching a specific target thanks to their membrane protein (Yang et al., 2018b). While the drug is encapsulated in extracellular vesicles, the advantage is that EVs act as a drug carrier, protecting the drug and transmitting it safely to the target site**.** Numerous studies have indicated that vesicles derived from mesenchymal stem cells (MSCs) appear especially useful for enhancing recovery after various injuries. As has been indicated in mice, injection of MSC-derived EVs suppressed hypoxia-induced inflammation and hypertension (Lee et al., 2012)**.** MSC EVs can exert a neuroprotective effect after a brain lesion (Xin et al., 2012)**.** Similarly, MSC EVs release miR-16 and other molecules to mouse breast cancer cells, exerting a decrease in vascular endothelial growth factor expression and a reduction in tumor growth (Lee et al., 2013)**.** More recently, a study has demonstrated that VEs derived from macrophages or liver sinusoidal cells treated with interferon-α deliver antiviral RNA and proteins to hepatocytes, thereby reducing hepatitis B virus replication (Li et al., 2013)**.** How different natural EVs promote these diverse responses remain to be elucidated. Drug loading into EVs can be achieved by these two strategies, either by directly loading the drug into exosomes or/and by loading the drug to target the mother cell during exosome biogenesis (Mittelbrunn and Sanchez-Madrid, 2010)**.** Moreover, in the case of lipophilic drug switching, the mechanism is relatively simple due to the interaction between the lipid bilayer of EVs and the drug, which occurs via a hydrophobic interaction (Shtam et al., 2013). Such methods as electroporation, incubation, sonication, and thawing have been performed for the exogenous loading of EVs with drugs (Jo et al., 2014).

The use of EVs as drug carriers presents several advantages but must conform to good manufacturing practice (GMP) (Chen et al., 2020). There are several important aspects of nanovesicle GMP. The low large-scale productivity of nanovesicles is one of the major problems encountered in the implementation of nanovesicle-based therapeutics; the main issues to be known about the various isolation methods are the physicochemical and purity properties of exosomes for the high-quality, uniquely shaped nanovesicle community and the standardization of storage requirements. The evolution of the therapeutic potential of nanovesicles by enriching them with therapeutic biomolecules is a simple way of improving their therapeutic potential (Abou-El-Enein et al., 2013). To use nanovesicles as reliable therapeutic agents, scalable manufacturing processes are needed to produce exosomes rapidly, cost-effectively, and reproducibly. In Table 6, we present a summary of another mammalian nanovesicle for therapeutic use.

**TABLE 6:** Therapeutic application of mammal-derived nanovesicles.

Mammal-derived nanovesicles source	Therapeutic application	Ref
Brain cells EVs	Plays a physiological and pathological role in the CNS.	András and Toborek (2015)
Human cardiac stromal cells	Improves cardiac ejection fraction, muscle strength, and exercise capacity	Aminzadeh et al. (2021)
Bovine breast milk-derived EVs	Transports RNA to the receptor cell. Immunoregulatory function	András and Toborek (2015)
Embryonic stem cells EVs	Enhances proliferation of human tissue	Burke et al. (2016)
Pancreatic EVs	Stimulates T and B cells	Cianciaruso et al. (2017)
Zebrafish osteoblasts-derived EVs	Involved in the maturation of osteoclasts	Kobayashi-Sun et al. (2020)
Plasma-derived EVs	Heals cardiac cells	O'Brien et al. (2020)
Astrocyte derived EVs	Neurodegeneration function	O'Brien et al. (2020)
Mesenchymal stromal cells derived EVs	Therapeutic function	Rohde et al. (2019)
Umbilical cord-derived EVs	Immunomodulatory function	Rohde et al. (2019)

## 6 Therapeutic efficacy of plant and mammalian EVs *in vitro* and *in vivo*

In this section, we will present the different studies that have been performed on plant and mammalian-derived extracellular vesicles *in vivo* and *in vitro*.

Studies that have evaluated plant-derived extracellular vesicles *in vivo* and *in vitro*: *in vitro* studies, researchers observed that Grapefruit-derived nano-vectors have important biocompatibility with little toxicity and apoptosis of macrophages and colon-26 cells *in vitro*, compared to commercial DC-Chol/DOPE liposome preparation (Nemati et al., 2022). Further *in vivo* and *in vitro* tests confirmed that Grapefruit-derived nano-vectors had the potential to regenerate mucus tissue in mice with colitis (Zhang et al., 2016b). Other studies have examined in detail the function of four plant-derived EVs (carrots, grapes, grapefruit, and ginger) on intercell communication. This study was realized *in vitro* and *in vivo*, and the results have suggested that these extracellular vesicles can regulate intestinal intercellular communication (Mu et al., 2014).

It was reported that grapefruit-derived nanovesicles were labeled with a lipophilic carbocyanine dye that permitted their *in vivo* tracking by fluorescence (Zhuang et al., 2016)**.** An *in vivo* study using diverse inflammation models in mice indicated that leukocyte-coated plant-derived extracellular vesicles (P-EVs) promoted the efficiency of Dox release at inflammatory sites (Li et al., 2018).

Studies that evaluated mammalian-derived extracellular vesicles *in vivo* and *in vitro*: *In vitro* and *in vivo* studies used to identify TGFβ receptors and mechanisms of action revealed pleiotropic roles for TGFβ in the control of pathophysiological processes. In addition, numerous preclinical results from *in vitro* cell models and *in vivo* animal models demonstrate the great potential of antitumor therapies using TGFβ-neutralizing antibodies and ligand traps that block the interaction of TGFβ with its receptors or selective receptor kinase inhibitors for the small molecule TGFβ (Liu et al., 2021). In another study, the effect of siponimod on ocular neovascularization *in vivo* was evaluated using suture-induced corneal neovascularization in albino rabbits. These results suggest that siponimod does not affect endothelial cell proliferation or metabolic activity but significantly inhibits endothelial cell migration, increases human microvascular endothelial cells’ (HMEC) barrier integrity, and reduces TNF-α-induced barrier disruption (Palette et al., 2013)**.** In another *in vivo* study, differences in protein cargo and non-coding RNA were identified that differentiated cardio-sphere-derived EV cells from mesenchymal stem cells and reflected differences in the effects of *in vivo* treatment *in vivo* (Walravens et al., 2021).

## 7 Toxicity and immunogenicity of mammal and plant-derived nanovesicles

The study of the efficacy of extracellular vesicles for immunogenicity or toxicity in therapy is fundamental to the preclinical development and development of their therapeutic properties. Evaluation of the toxicity and security of EVs *in vitro* and *in vivo* will permit the establishment of the dose for future clinical use and the recommendations for safe consumption for human application. An ideal drug delivery system should guarantee non-toxicity and non-immunogenicity without secondary effects both *in vitro* and *in vivo* (Zhuang et al., 2015). However, there are still a few tentative discoveries of their cytotoxic effect on living subjects. So far, plant-derived nanovesicles have indicated fascinating biocompatibility due to their natural origin. To evaluate the toxicity of plant-derived nanovesicles’ toxicity, the authors used tumor-targeted Grapefruit-derived nano-vectors (GDNV) and examined whether significant tissue damage occurred in the organs. Because of the effectiveness of GDNV on tumors, GDNV accumulates less in the spleen and liver; this permits the minimization of systemic drug toxicity to normal tissues while improving blood diffusion of delivered drugs. Histological analysis of the heart, liver, spleen, lungs, or kidneys has not demonstrated significant damage compared to the control group, indicating that plant-derived nanovesicles can be applied as nano-drug delivery platforms, improving drug efficacy and reducing potential toxicity (Kim et al., 2022). In a recent study, markers such as pro-inflammatory cytokines and serum levels of liver enzymes, including alanine aminotransferase (ALT) and aspartate aminotransferase (AST), have been evaluated to determine the potential cytotoxic effects of grape-derived nanovesicles in mice. Mice were pretreated with grapefruit-derived nanovesicles and commercially available DOTAP (1,2-diammonium-propane)-DOPE (dioleoyl phosphatidylethanolamine) liposomes.

Loyal-3-trimethylammonium-propane)-DOPE (dioleoyl phosphatidylethanolamine) liposomes and pro-inflammatory cytokines were significantly elevated in liposomes. In addition, the authors found that no increase was recorded in the group of mice treated with Plant Exosome-like Nanovesicles (PELNV). Furthermore, no pathological alterations were observed in histological samples of the liver, kidney, spleen, and lung from mice treated with plant exosome-like nanovesicles (Ding et al., 2014).

In addition, in a study, researchers evaluated the toxicity of milk-derived EVs. The result indicated that no systematic toxicity or immunogenicity was observed. In the experiment, animals were given an intravenous injection containing milk-derived EVs. The results of the blood tests confirmed no damage from these EVs, and no markers of harm or toxicity to the kidneys or liver were found (András and Toborek, 2015). Another study conducted *in vitro* to evaluate the toxicity of EVs derived from mesenchymal cells and bovine milk indicated that there was no genotoxic response to these two EVs, but a platelet aggregation induced by collagen in a dose-dependent manner was recorded. Another study was conducted *in vitro* to evaluate the safety of HEK293T cell-derived EVs. Mice were injected intravenously with HEK293T cell-derived EVs. The results indicated no toxic effects, no immune changes, and no alterations in EVs (Rohde et al., 2019).

In effect, plant- and mammal-derived nanovesicles present numerous advantages in terms of biocompatibility, stability, biodistribution, and cellular internalization. However, some challenges related to biosafety and toxicity may also be encountered due to unknown bioactive components of the plant.

## 8 Plant-derived EVs in clinical trials

Based on the results of the effects of plant-derived nanovesicles, many clinical trials have been initiated to complement the results (Table 7). In another clinical trial (NCT01668849), grape-derived nanovesicles were administered as an anti-inflammatory agent to diminish oral mucositis in head and neck cancer patients undergoing chemotherapy (Xie et al., 2020; Nemati et al., 2022)**.** Trials were conducted after 6–7 weeks of treatment. In addition, another clinical trial (No. NCT01294072) was carried out in 2011 on turmeric-derived nanovesicles for more effective delivery of curcumin to the gut. (Xie et al., 2020). The effect of turmeric-derived nanovesicles on malignant and normal clonal cells and their effect on the immune system were applied to colon cancer patients. This study was developed to investigate the effect of turmeric nanovesicle administration on oral tablets under recruitment conditions (Xie et al., 2020).

**TABLE 7:** Plant-derived EVs in clinical trials.

Condition/Disease	Year/Phase	EV-derived	Therapeutic molecule	Administration	Results/Status	Ref
Oral-Mucositis (NCT01668849)	Phase I 2012	Grapes	Not determined	Dietary Supplement oral, daily for 35 days	Active, not Recruiting	Redman (2012)
Colon cancer (NCT01294072)	Phase I 2011	Plants	Curcumin	Tablets oral, daily for 7 days	Active, not Recruiting	Wu et al. (2017)
Insulin-related conditions Chronic inflammation in Polycystic ovary syndrome patients (NCT03493984)	2018 Preliminary	Ginger Aloe	Not determined	Not determined	Recruiting	PCOS (2021)

## 9 Mammalian-derived EVs in clinical trials

There were numerous clinical trials on mammalian-derived extracellular vesicles for therapeutic purposes. The application, dose, number of patients, identification number, and follow-up are indicated. Currently, studies are in clinical trials (listed at www.clinicaltrials.gov).

In a study, the application of mesenchymal stem cell (MSC)-derived exosomes based on siRNA-exosome therapy is used in the treatment of pancreatic cancer patients with KrasG12D mutation via KrasG12D in a phase I clinical trial (ClinicalTrials.gov identifier: NCT03608631). Despite these encouraging results for the application of MSC-derived exosomes as drug vesicles for cancer treatment in the clinic, many challenges remain (Mendt et al., 2018)**.** A combination of ascites-derived exosomes with granulocyte-macrophage colony-stimulating factor (GM-CSF) was tested in a phase I clinical trial for the treatment of advanced colorectal cancer, which was found to be feasible and safe and also capable of eliciting more CTL infiltration in tumor regions (Dai et al., 2008)**.** A study was performed at Sahel University Hospital, Cairo University, to evaluate the effect of consecutive doses of CSM-EV in 20 patients with type 1 diabetes, with a follow-up of 3 months. The results are not yet available (Mendt et al., 2018)**.** In the same hospital, another study recruited 20 patients with chronic renal failure and were administered two doses of CMS-EV from the umbilical cord with follow-up for 1 year and the results are already available (Nassar et al., 2016)**.** Finally, a clinical trial, which involves the injection of MSC-EVs engineered with miR-124 for the treatment of patients after acute ischemic stroke, was approved in Iran (Grange et al., 2019) (see Table 8).

**TABLE 8:** Mammalian-derived EVs in clinical trials.

Diseases and identification number	Number of patients	EV-derived	Location	Administration	Results/Status	Ref
Cerebrovascular disorders acute ischemic stroke (NCT03384433)	5	mesenchymal stem cells (MSC)	Shahid Beheshti University of Medical Sciences, Teheran Iran	Allogenic MSC-EVs enriched by miR-124	Not yet recruiting	Grange et al. (2019)
Pancreatic cancer with KrasG12D mutation via KrasG12(NCT03608631)	Not determined	MSC	Not determined	Not determined	Phase I	Mendt et al. (2018)
Diabetes mellitus type 1 (NCT02138331) 20		MSC	Sahel Teaching Hospital Sahel, Cairo, Egypt	Two doses of CSM-VE	Not Recruiting	Nassar et al. (2016)
Chronic renal disease	20	MSC	Sahel Teaching Hospital Sahel, Cairo, Egypt	Two doses of umbilical cord MSC-EV (100 μg/kg/dose)	Concluded	Nassar et al. (2016)

## 10 New therapeutic approaches to extracellular vesicles

Therapies based on plant- and mammalian-derived extracellular vesicles (EVs) are attracting growing interest as a promising therapeutic approach in various medical fields for the treatment of diverse diseases. The benefits of these devices include their ability to cross biological barriers, enhance drug pharmacokinetics and therapeutic efficacy, and diminish the toxic side effects commonly associated with conventional synthetic nanovesicles. In addition, EVs can be chemically modified to incorporate additional ligands for targeted drug delivery. In addition to their ability to transport biomolecules to other cells, namely, proteins, lipids, and nucleic acids, they are a promising tool for clinical diagnostic and disease prognostic assessments. However, many technological, functional, and safety aspects still need to be taken into account (Li et al., 2023b; Sadeghi et al., 2023)**.**

Targeted drug therapy: EVs can be modified to express specific surface ligands, giving them the ability to selectively target particular cells or tissues. This offers exciting opportunities for targeted drug delivery while at the same time minimizing adverse effects on other healthy tissues. However, the obstacle to EV therapy that needs to be overcome to achieve a therapeutic result is the regulation of EV uptake. Many steps have been taken to improve the factors influencing EV uptake, including cell source selection, cell growth procedures, extraction and purification methods, storage, and routes of administration. Rapid distribution, targeted delivery, and non-targeting of EVs are current challenges that need to be addressed (Claridge et al., 2021; Esmaeili et al., 2022)**.**

Gene therapy: Gene therapy can be applied to treat genetic diseases, control gene expression in particular cells, or trigger specific healing processes. In addition, there is an urgent need for drug delivery vectors capable of efficiently transferring therapeutic cargo to recipient cells while bypassing the cellular barriers created by the development of new genotoxic anti-cancer therapies. Drug delivery methods have been proposed to overcome these restrictions, but their successful clinical application has been thwarted by the occurrence of unanticipated adverse effects and related toxicities (Duan et al., 2021; Jayasinghe et al., 2021)**.**

Immunotherapy: the use of extracellular vesicles has been applied to the field of cancer immunotherapy, and this has made remarkable progress, becoming a real tool in the fight against cancer. Extracellular vesicles were also seen as vectors for molecules capable of activating an immune response and destroying cancer cells. Based on this observation, the new approach is to use extracellular vesicles as a new means against cancer. The immunotherapeutic approach based on extracellular vesicles in the treatment of cancer patients has been demonstrated, even in the case of advanced cancers. The use of extracellular vesicles in immunotherapy does not reveal any qualitative or moral difficulties. Progress in this field will therefore lead to practical results and a new, innovative approach to the fight against cancer. (Giacobino et al., 2021; Marar et al., 2021).

Tissue regeneration: A promising approach in regenerative medicine is the therapeutic use of extracellular vesicles for tissue regeneration. Extracellular vesicles can contain growth factors and proteins essential to the tissue regeneration process. Growth factors can be selectively delivered to target cells via these vesicles, promoting their proliferation and differentiation. This approach is still in the development stage, so it will be some time before it becomes established as a clinical therapy for tissue regeneration. It could 1 day provide new therapeutic alternatives for the treatment of various diseases and conditions associated with tissue damage. (Ju et al., 2022; Zheng et al., 2022).

## 11 Conclusion and prospects

In recent decades, VEs had capabilities of cell-to-cell communication in prokaryotes and eukaryotes due to their ability to transfer active biomolecules such as proteins, lipids, nucleic acids, and other biologically active substances, and have considerable roles in several physiological and pathological mechanisms. In recent years, extracellular vesicles present numerous benefits in terms of biocompatibility, therapeutic capacity, targeting ability, and cellular uptake. Their low toxicity and immunogenicity make extracellular vesicles an emerging, versatile, and promising biotherapy for a very wide variety of diseases.

In addition, research has indicated that EVs are essential for communication between plants, mammals, and pathogens and that they perform significant roles in a variety of pathologies. However, despite the constant progress of discoveries, complementary studies on extracellular vesicles are still needed in several aspects, especially cellular and molecular biogenesis, functions, and uptake which are not yet well known. In the context of isolation, we remark that particularly for plant-derived EVs, the results obtained in the various studies often vary according to plant derivative, the isolation technique, or the physiological state of the plant, as well as the content of the isolated nanoparticles due to the lack of a standardized isolation protocol. Also, improvements in EV isolation permit the fabrication of high-purity EVs for biomarker discoveries. As new markers of EV subcategories continue to emerge, the capacity to use high-fluorescence microscopy should improve the understanding of EV subcategory biogenesis. In addition, there is a lack of information on plant-derived EV biogenesis, as there are few specific protein markers for EVs, and determining the biological characterization of exosomes needs further investigation, as the surface markers and other characteristic elements of plant exosomes remain uncertain. The application of extracellular vesicles’ therapeutic potential requires further clinical trials to obtain more precise information on results, development of effective isolation, scale-up methodologies, stability, and properties of either plant- or mammalian-derived extracellular vesicles. Although clinical trials on some extracellular vesicles are underway, the regulatory aspects of their use as therapeutic agents are not known. Despite the difficulties and obstacles encountered by researchers in this field, it has been proven that extracellular vesicles have natural therapeutic capacity advantages without toxicity or side effects. Therefore, when targeted and developed with multidisciplinary expertise, EVs can be transformed into reusable therapeutic agents to fight against various pathologies.

The advantage of using nanovesicles is that the efficacy of natural products (plants or mammals) in therapeutic applications as indicated in these studies can be improved by increasing their bioavailability. In systems, nano-distribution can also be used to overcome the limits of therapeutic applications of natural products for several reasons: their capacity to target nanovesicles to specific organs, thus improving selectivity, therapeutic application, efficacy, and security. Nanovesicles passively target pathological sites of action without the addition of specific ligand fragments. The therapeutic efficacy of nanovesicles can reduce side effects because of their properties. Nanovesicles increase the solubility of natural compounds. Nanovesicles can dissolve rapidly in the blood, so they appear to be able to administer small-sized drugs. In addition, it is well established in these studies that the application of nanovesicles in clinical trials is an ideal candidate for the treatment of many diseases. The authors have indicated that several preclinical experiments have confirmed the advantages of nanovesicles for the treatment of numerous pathologies ranging from regenerative medicine to cancers.

Ultimately, we expect that with further research on all the aspects we have just mentioned above on the use of extracellular vesicles, in the next few years, we will probably see an increase in the use of extracellular vesicles both for the diagnosis against more widespread pathologies and as a starting point for the development of new therapies.
